# T Lymphocytes: A Promising Immunotherapeutic Target for Pancreatitis and Pancreatic Cancer?

**DOI:** 10.3389/fonc.2020.00382

**Published:** 2020-03-24

**Authors:** Qi Zhou, Xufeng Tao, Shilin Xia, Fangyue Guo, Chen Pan, Hong Xiang, Dong Shang

**Affiliations:** ^1^Laboratory of Integrative Medicine, The First Affiliated Hospital of Dalian Medical University, Dalian, China; ^2^Institute (College) of Integrative Medicine, Dalian Medical University, Dalian, China; ^3^School of Chemical Engineering, Dalian University of Technology, Dalian, China; ^4^Department of General Surgery, Pancreatic-Biliary Center, The First Affiliated Hospital of Dalian Medical University, Dalian, China

**Keywords:** T lymphocyte, acute pancreatitis, chronic pancreatitis, pancreatic cancer, immunotherapy

## Abstract

Pancreatic disorders cause a broad spectrum of clinical diseases, mainly including acute and chronic pancreatitis and pancreatic cancer, and are associated with high global rates of morbidity and mortality. Unfortunately, the pathogenesis of pancreatic disease remains obscure, and there is a lack of specific treatments. T lymphocytes (T cells) play a vital role in the adaptive immune systems of multicellular organisms. During pancreatic disease development, local imbalances in T-cell subsets in inflammatory and tumor environments and the circulation have been observed. Furthermore, agents targeting T cells have been shown to reverse the natural course of pancreatic diseases. In this review, we have discussed the clinical relevance of T-cell alterations as a potential outcome predictor and the underlying mechanisms, as well as the present status of immunotherapy targeting T cells in pancreatitis and neoplasms. The breakthrough findings summarized in this review have important implications for innovative drug development and the prospective use of immunotherapy for pancreatitis and pancreatic cancer.

## Introduction

Pancreatic disease is the most common gastrointestinal disease, and is defined as ongoing or chronic inflammation, immune dysfunction, and an uncontrollable inflammatory malignant transformation of the pancreas (1, 2). Acute pancreatitis (AP) is associated with a high risk of severe morbidity characterized by an acinar cell injury and exocrine abnormalities. This hyperinflammatory process is accompanied by an infiltration of the innate immune cells (mainly macrophages and neutrophils), which can provoke the systemic inflammatory response syndrome (SIRS) and even the multiorgan dysfunction syndrome. The next phase is the compensatory anti-inflammatory response syndrome, in which T cells have been considered to play a protective role against hyperinflammation (3). If the inflammatory instigators remain active, the pancreas undergoes a persistent and an irreversible fibrotic reaction, typical of a chronic pancreatitis (CP) (4, 5). According to a 2019 report, the estimated number of annual outpatient visits for pancreatitis in the USA is 757,161. Pancreatitis has the fourth highest 30-day readmission rate among pancreatic conditions and accounts for $2,386 million of the total healthcare expenditures (6). In addition, pancreatic inflammation is considered a key mediator and a long-term risk factor for the development of pancreatic cancer (PC), which currently is the fourth most fatal malignancy and has been predicted to become the second deadliest cancer in the USA and Europe by 2030 (7, 8). PC has a broad spectrum of neoplasms, including non-ductal tumors, pancreatic ductal adenocarcinoma (PDAC) and its classical precursor lesions (pancreatic intraepithelial neoplasia (PanIN), intraductal papillary mucinous neoplasms (IPMNs), and mucinous cystic neoplasms (9). Non-ductal pancreatic tumors, such as neuroendocrine neoplasms and acinar cell carcinomas, are rare. The former involve a well-differentiated pancreatic neuroendocrine tumor and a poorly differentiated neuroendocrine carcinoma (10, 11). Owing to the complicated pathology and multifactorial features of pancreatic diseases, there is a lack of efficient treatments and Food and Drug Administration-approved therapies for these diseases (12, 13).

T cells encompass several subsets with distinct functions, including the T helper cells (Th cells), the cytotoxic T (T_C_) cells, the regulatory T cells (Tregs), the memory T cells, the natural killer T cells, and the gamma delta (γδ) T cells (14). While neutrophils and monocytes/macrophages have been hypothesized to be the major leukocyte populations infiltrating the inflamed pancreas, local imbalances in T cells in the inflammatory sites and in circulation have been observed in pancreatitis (15), suggesting that T cells may also have a prominent effect on the progression of pancreatitis (16, 17). Simultaneously, intratumoral T cells are heterogeneous, and strong effector T-cell infiltration is associated with a prolonged survival, which provides a rationale for the use of immunotherapy in PC (18, 19). In the present review, we have discussed the clinical relevance of T-cell alterations in pancreatic diseases and the underlying mechanisms, and we have provided evidence supporting the potential of immunotherapeutic strategies to regulate T cells in these diseases.

## Alteration of T Cells in Pancreatic Disease

### CD4+ T Cells

Recent extensive evidence shows a substantial reduction and an impaired activity of the peripheral CD4+ T cells in the human AP (20, 21). Moreover, there is a close relationship between a decreased CD4+ T-cell population in the circulation and complications such as local necrosis, SIRS, and persistent organ failure during severe acute pancreatitis (SAP). Both a prospective survey and a retrospective study revealed that a reduction in the peripheral CD4+ T cells at the onset of AP is a simple, early, and an accurate parameter for predicting the clinical outcomes of AP or progression to persistent organ failure, with 61.54% sensitivity and 90% specificity (22, 23). The apoptotic rate of the CD4+ T cells is positively correlated with the number of days to resolution of SIRS in the event of AP, but not with late resolution of SIRS (24). Furthermore, Liu et al. observed that a sustained low level of the peripheral CD4+ T cells is a potential predictor of the abdominal compartment syndrome in SAP. However, the sensitized CD4+ T cells migrate to the inflammatory sites, leading to a significantly increased CD4+ T-cell count in the pancreas during AP (25). These findings indicate that an early abnormal number of CD4+ T cells increases the severity of pancreatic injury through an intrapancreatic infiltration and the release of proinflammatory cytokines. More multicenter clinical studies will be needed to confirm the sensitivity and specificity of changes in the peripheral CD4+ T cells for the diagnosis of complications or classification in AP patients.

A clinical study comparing the alterations in the peripheral immunocompetent blood cells during CP and after pancreatic head resection revealed that the populations of CD3+ T cells and circulatory CD4+ T cells were markedly expanded in patients with CP (26). In contrast, elimination of the chronic inflammation via pancreatic head resection restores the distribution and function of the T cells. It is well established that the recruitment of CD4+ cells into pancreatic lesions by local chemokines is implicated in the pathogenesis of CP (27, 28). Nakayama et al. failed to induce pancreatitis in the severe combined immunodeficient mice by administering alcohol and lipopolysaccharide (LPS) until an intraperitoneal injection of splenocytes (CD4+ or CD8+ T cells obtained from the wild-type mice), which showed that acquired immunity was essential for disease development besides repeated stimulation of the innate immune system (29). A basic study showed the evident role of T cells in exacerbating a CP progression and provided a pharmacological basis for the targeting of T cells as a novel therapy. Whether such a treatment will be curative remains to be evaluated in further laboratory and clinical studies.

Autoimmune pancreatitis (AIP) is a peculiar form of CP with a poorly understood autoimmune etiology that accounts for 5−6% of all the CP cases (30, 31). Accumulating evidence suggests that autoreactive T cells are key players in the pathogenesis of AIP (32). Increased numbers of the activated CD4+ and CD8+ T cells have been detected in the peripheral blood lymphocytes and the pancreas of AIP patients (33). Noteworthy, the CD4+ T cells, rather than the CD8+ T cells, have been hypothesized to be critical and sufficient for an AIP pathogenesis (34). Adoptively transferred CD3+ T cells and the CD4^+^CD44^high^ memory T cells collected from sick mice with AIP efficiently induced AIP in the susceptible MRL/MpJ recipient mice (35). Moreover, adoptive transfer of CD4+ T cells derived from the syngeneic mice with AIP induced pancreatitis in the recipient RAG2-deficient mice, suggesting that autoreactive CD4+ T cells may induce autoimmunity in the pancreas (36). These findings suggest that CD4+ T cells may play a predominant role in AIP.

**Figure 1:**
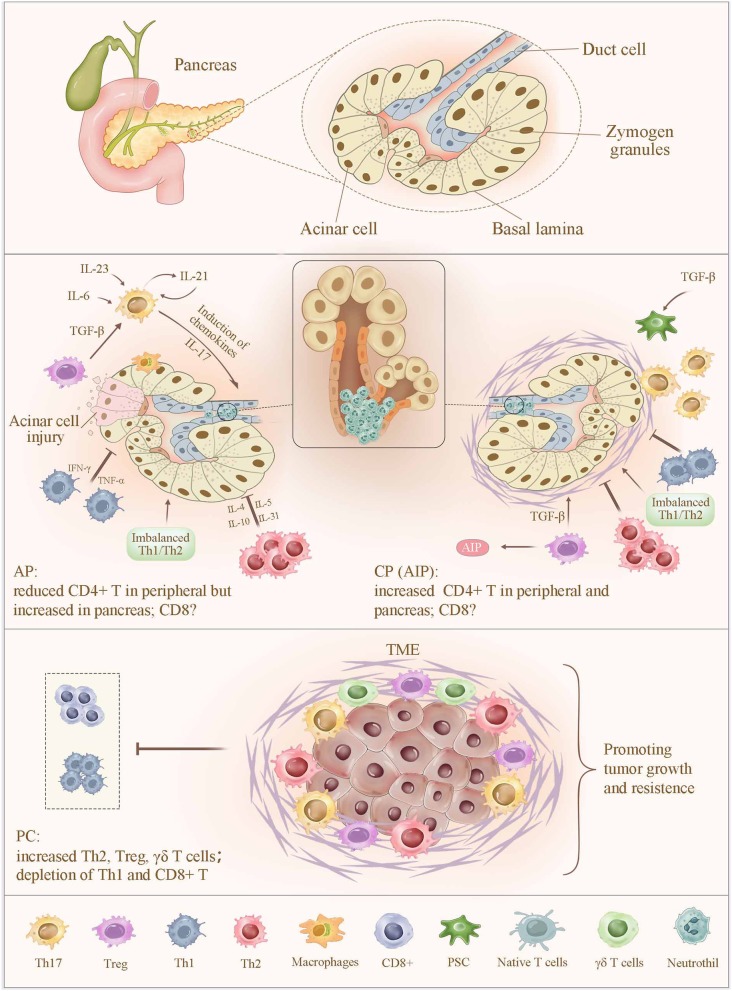
Alteration of T cells in pancreatitis and pancreatic cancer. In AP, the sensitized CD4+ T cells migrate to the inflammatory sites, leading to a significantly increased CD4+ T-cell count in the pancreas and a sustained lower level of peripheral CD4+ T cells. In contrast, an increased number of CD4+ T in peripheral and pancreas were observed in CP. Abnormal expression of CD4+ T cells increases the severity of pancreatic injury by intrapancreatic infiltration and the release of proinflammatory cytokines. Moreover, imbalance of Th1/Th2 cells, together with a reduced number and impaired function of Tregs, promotes pancreatic inflammation. Th17 cell is capable of amplifying the inflammatory cascade and pancreatic damage via regulating the expression of inflammatory molecules and chemokines, as well as inducing the neutrophil chemoattraction to the secretory ducts of the pancreas and the subsequent formation of aggregated neutrophils hampers the secretory flow and induces a focal pancreatitis due to ductal occlusion, which strongly determines the severity of AP and CP. In PC, infiltrating immune cells, including Th2, Treg, and γδ T cells, contribute to a more favorable TME to suppress the CD8+ T_C_-cell infiltration and subvert an immune surveillance, thereby supporting tumor proliferation, invasion, and metastasis. AP, acute pancreatitis; CP, chronic pancreatitis; TME, tumor microenvironment.

PC is resistant to immunotherapy because of various immunosuppressive mechanisms, including its low immunogenicity and a non-inflamed phenotype (37). CD4+ T cells account for up to 5% of the total intratumoral cells (38). Analysis of the immune landscape of the tumor microenvironment (TME) by single-cell RNA sequencing revealed that T_C_ cells and the activated Th cells were highly prevalent in low-grade IPMNs, but were gradually exhausted during the multistep progression of IPMNs to PDAC (39). The majority of neuroendocrine pancreatic tumors show a robust intratumoral immune response, comprising CD3+, CD4+, and CD8+ T cells (40). These cells, which mediate protumor effects and immunotherapy resistance, continue to attract increasing research attention (Figure 1).

### Subsets of CD4+ T Cells

CD4+ T cells can differentiate into various specialized lineages: Th1, Th2, Th9, Th17, Treg, and T follicular helper (Tfh) cells (41). Generally, different types of CD4+ T cells counterbalance each other to maintain an immune homeostasis. Nevertheless, an imbalance in the CD4+ T-cell subsets caused by a hyperactivation of any subset in a functional disruption of the immune system can lead to various diseases, such as inflammatory, autoimmune, or even cancer, because of an inefficient pathogen clearance (42).

#### Th1/Th2 Cells

Over the course of AP, Th1/Th2 imbalances are dynamic, with a shift from Th1 to Th2 cells (43). This decrease in the Th1/Th2 ratio is likely due to an early (within one week) suppression of Th1 cells and up-regulation of Th2 cells. However, over time, the production of Th1 cells and the Th1/Th2 ratio strongly increase, whereas the Th2 production gradually decreases in the periphery (44–46), suggesting that both the shift from Th1 cell suppression toward a Th2 cell response and the serious imbalance in Th1/Th2 cells may be responsible for the induction of an inflammatory disease. Rodriguez et al. measured serum concentrations of Th1 and Th2 cell cytokines to acquire objective markers for evaluating the severity of AP and predicting its course, and found that a Th1 cytokine profile is closely related with SAP, whereas a Th2 profile is closely related with moderate AP or moderately severe AP (47). Moreover, immune profiling of the lymphoid tissue surrounding the pancreas revealed a substantial anti-inflammatory response driven by the Tregs and the Th2 cells (3). Therefore, reducing the Th1/Th2 ratio and maintaining their balance may be a strategy to prevent further deterioration in AP.

Jupp et al. found significant increases in the Th1, Th2, and Th17 cell populations, without polarization of the Th cell response toward either a Th1 or a Th2 phenotype, in the peripheral blood in CP, whereas Th1 and Th17 cells predominantly infiltrated the pancreas, without evident Th2 cell involvement (48). Furthermore, the transition of chronic fibroinflammatory responses to a protumorigenic effect is mediated by the suppression of the myeloid differentiation factor 88, attributed to the dendritic cell–Th2 axis, which expands the polarization of the intrapancreatic Th2 cells and inhibits the differentiation of other Th-cell subsets and CD8+ cells (49). Thus, decreasing the proportion of Th1 cells has been considered to alleviate inflammation, and reducing the number of Th2 cells may partially inhibit the transformation of inflammation into cancer.

It is generally believed that the relative preponderance of Th1 and Th2 cells is an important pathophysiological factor in various autoimmune diseases. However, there is a discrepancy regarding the predominance of different T-cell subsets in the progression of AIP. It has been demonstrated that Th1 cells producing interferon (IFN)-γ and peripheral blood IFN-γ were evidently higher in AIP patients than those in controls, whereas IL-4 levels did not differ (50), showing that immune responses of Th1 cells were more activated than those of Th2 cells. In addition, treatment with IFN-γ was deleterious for AIP in mice (51, 52), suggesting that Th1 cells promoted an AIP development. In contrast, Th2 (IL-4, IL-5, and IL-13) and regulatory (IL-10 and TGF-β) cytokines significantly infiltrated the affected tissues in patients with the IgG4-related sclerosing pancreatitis and cholangitis, showing that the immune reaction predominantly was mediated by the Th2 cells (53). A reasonable interpretation for these conflicting findings is that both the Th1 and the Th2 cell immune responses occur in the development of AIP, and the Th1/Th2 cell ratio may shift dynamically between the early and advanced stages of AIP. For example, Okazaki et al. suggested that Th1 cytokines may be essential in the induction of AIP, whereas Th2 cytokines play a vital role in disease progression (54).

The tumor stroma in pancreatic carcinoma is predominantly infiltrated by Th2 rather than Th1 cells (38), which is an independent predictive marker of poor survival. The Th2/Th1 cell ratio in the infiltrate of the tumor immune response was significantly correlated with reduced survival after a surgical resection, pointing to the importance of the balance between Th2 and Th1 cells in the TME in clinical outcomes (55). Further, these findings suggest that the Th1-polarized CD4+ T cells may mediate tumor protection and may be associated with prolonged survival in PC.

#### Th17 Cells

Th17 cells are the main source of the hallmark cytokine IL-17, which exhibits a positive correlation with disease severity and represents a valuable prognostic factor for evaluating disease severity in patients with AP (56). It has been suggested that IL-17 is capable of amplifying the inflammatory cascade and pancreatic damage via regulating the expression of inflammatory molecules and chemokines, as well as recruiting neutrophils and macrophages to the site of injury/inflammation during the pathogenesis of AP *in vivo* and *in vitro* (45, 57). Although the proinflammatory Th17 pathway has been shown to initiate an early SIRS in AP, IL-17A is not responsible for the second hit (58, 59). The second hit is initiated by systemic sepsis arising from a serious impairment of the intestinal barrier function and the gut-derived infection following SIRS, and in this phase, organ dysfunction and even death can occur (60). Thus, Th17 cells are required for the induction of pancreatitis, suggesting that therapeutic modulation of Th17 cells may ameliorate the pancreatic inflammation.

In CP, there is a much higher magnitude of the Th17 cell elevation. The underlying mechanism involves the transcriptional repression of Bach2 (BTB and CNC homology basic leucine zipper transcription factor 2), an important regulator of the T cell-mediated immune homeostasis that mediates inflammation by inducing the polarization of the pathogenic Th17 cells in CP (61). IL-17A induces the neutrophil chemoattraction to the secretory ducts of the pancreas, and the subsequent formation of aggregated neutrophils hampers the secretory flow and induces a focal pancreatitis due to ductal occlusion, which strongly determines the severity of CP (62). In a comparison of the type 1 and the type 2 AIP, Th17-cell infiltrates were significantly more pronounced in the periductal compartment of the type 2 AIP, which was induced via neutrophil recruitment by both IL-17A and the induction of the granulocyte-macrophage colony-stimulating factor secretion, resulting in partial ductal destruction (63).

Besides IL-17, Th17 cells also produce signature cytokines, including IL-21, IL-22, and IL-23. Circulatory IL-21 is transiently elevated during the second hit of AP, which may potentiate an immune imbalance and immune paresis (64). IL-21 worsens inflammatory disease by inhibiting the Tregs, and loss of the IL-21/IL-21R signaling in Il2^−/−^ Il21r^−/−^ mice reduces the population of Th17 cells, suggesting the critical role of the IL-21/IL-21R signaling in Th1-cell generation, differentiation, and survival (65). IL-22 belongs to the IL-10 cytokine family and has been recognized to be important in antimicrobial defense, regeneration, and protection against damage (61). IL-22 plays a protective role in pancreatic inflammation by up-regulating the expression of anti-apoptosis genes (*Bcl-2* and *Bcl-XL*), suppressing the autophagic pathway, and reducing the formation of an autophagosome (66, 67). Aryl hydrocarbon receptor is a ubiquitously expressed transcription factor that promotes pancreatic IL-22 production upon activation (68). IL-22 induced by cigarette smoke containing aryl hydrocarbon receptor agonists promotes a CP development by increasing the deposition of the extracellular matrix via activation of the IL-22 receptor A1, which phosphorylates the signal transducer and activator of transcription (STAT) 3 in the pancreatic stellate cells (69). The interaction between IL-23 and IL-23R has numerous biological effects, including the promotion of memory T-cell proliferation, Th17-cell differentiation, and IL-17 secretion, in immune-related diseases (70). While the cardinal effects of IL-23 in pancreatitis remain to be fully elucidated, a study *in vivo* revealed that IL-23 is strongly expressed in the pancreas and administration of an exogenous recombinant IL-23 promoted the coxsackievirus B3 infection-induced pancreatitis (71). Thus, the cytokine milieu of Th17 cells is an interesting topic for future research.

The roles of Th17 cells in PC remain controversial as both pro- and antitumorigenic effects have been observed, possibly due to differences in the model establishment. However, the functions of Th17 cells are primarily mediated by IL-17. Using a murine model of PanIN, McAllister et al. found that the oncogenic Kras induces Th17-cell infiltration and that IL-17 overexpression dramatically drives tumor initiation and progression (72). IL-17 is expressed in the TME and exerts protumorigenic effects through complex mechanisms involving cross-talk among the γδ T cells, myeloid-derived suppressor cells, and tumor cells (73). Moreover, immune cell-derived IL-17 was shown to induce stem-cell features in PC cells, contributing to the initiation and progression of PanIN (74). A clinical study has revealed that overexpression of the IL-17 receptor is strongly related to a postoperative metastasis and a poor progression in PC patients and that genetic or pharmacologic blockade of IL-17 has antitumor effects (75). In contrast, Th17-cell infiltrates in the subcutaneous murine PC tumors (Pan02) exert an antitumor effect through delaying the tumor growth and survival, which is partly attributed to the fact that certain cytokines in the TME could reverse the tumor-associated immune suppression. For example, IL-6 has the ability to suppress Treg development and induce the Th17 cells in the presence of TGF-β (76). Emerging technologies, such as single-cell sequencing, are expected to soon reveal the exact roles of Th17 cells in PC.

#### Tregs

Tregs mediate the control of the inflammatory response after a serious injury in SAP (77). An elevated percentage of the circulating CD4+CD25+CD127^low/neg^ Tregs reportedly was associated with an increased risk of infected necrosis and mortality (78). By contrast, level of the peripheral CD4+CD25+CD127^high^ Tregs showed a significant negative correlation with multiple organ failure in the early stage of AP (79), implying that activated effector T cells phenotyped as CD4+CD25+CD127 ^high^ may be an independent prognostic biomarker for SAP. One hypothesis that explains the progression of AP to CP attributes the major cause to the failure of Tregs in inhibiting the effector T cells, resulting in a balance disruption (80).

Unlike the dysregulation of Tregs in other human autoimmune diseases (81), in AIP, Tregs are likely activated and critical for maintaining self-tolerance, which is dependent on Foxp3, TGF-β, and IL-10, through their inhibitory effects on the effector T cells (50). AIP has been identified as a T cell-driven disease by blockage of the T_C_ cell-associated protein 4 (a potent attenuator of T-cell responses) in MRL/MpJ mice (a model of spontaneous AIP). In these mice, Tregs are suppressed and the effector T-cell response is increased, leading to a worsened severity of AIP accompanied by pronounced organ destruction and an infiltration of the inflammatory cells (32). In patients with type 1 AIP, high levels of autoantibodies exist against lactoferrin, carbonic anhydrase II, and pancreatic trypsin inhibitor and induce the peripheral Tregs and cause the activation of both Th1- and Th2-type immune cells. Besides fibrosis induced by TGF-β, oversecretion of IL-10 from the costimulator-positive Tregs can stimulate IgG4 production in the peripheral B cells and the IgG4-positive cell infiltration in the pancreatic lesion (82). Thus, an increase in Tregs may influence IgG4 production, and a decrease in naive Tregs in the periphery may promote AIP development. Furthermore, significant Treg infiltration has been observed in the major duodenal papilla through an endoscopic biopsy and has been found to be reliable for the differential diagnosis of AIP and PC, with an extremely high specificity (100%) for AIP (83).

Tregs potently expand by nearly 2-fold in the peripheral blood (84) and are recruited early in the course of PC, and ultimately account for 20−25% of the intratumoral CD4+ T cells (85). Their recruitment is partly mediated through the chemotaxis of an overexpressed chemokine receptor type 5, which induces the Treg homing and activation (86, 87). An increased number of Tregs has been hypothesized to contribute to the T-cell inhibition via an increased production of the immunosuppressive molecules, such as TGF-β and IL-10 (86). Additionally, PC cells directly secrete IL-10 and TGF-β, which decrease the activity of the antigen-presenting dendritic cells and inhibit the T_C_-cell function while promoting Treg differentiation (88, 89). Expanded populations of both the peripheral and the intratumoral Tregs have been observed in IPMNs, and high Treg levels have shown a predictive value in the progression and multistep carcinogenesis of PC (90–94). The positive correlation between Tregs and indoleamine 2,3-dioxygenase reflects the pathological aggressiveness of IPMNs, which is determined by the Notch signaling (95). Downs-Canner et al. observed a clear tumor-associated Th17-to-Treg cell conversion, which served as an alternative source for Tregs in PC (96). In K-rasLSL.G12D/+; Trp53R172H/+; Pdx-1-Cre (KPC) model mice, depletion of Tregs suppressed the progression of early-stage PanINs by decreasing the Treg population in the pancreatic lymph nodes and increasing the IL-17/IFN-γ-secreting CD4+ T cells (97). In a study of 101 patients with pancreatic neuroendocrine tumors, the number of Tregs was identified as an independent prognostic factor and was negatively correlated with the overall survival (98). Based on a comparison between patients with pancreatic neuroendocrine tumors with or without liver metastases, Katz et al. found that high levels of intratumoral T cells were positively correlated with an improved recurrence-free survival in patients without metastases, whereas the presence of dense Tregs predicted a short overall survival in those with metastases (99). Thus, a high prevalence of Tregs is an independent marker of poor prognosis in patients with PC. Whether reducing the number of Tregs will influence tumor progression remains to be explored, but may be a promising direction.

#### Th9/Tfh Cells

Th9 cells, a newly discovered subset of the proinflammatory CD4+ T cells, share commonalities with Th2 cells, but drive inflammation in autoimmune diseases and allergic inflammation (45). Th9 cells secrete IL-9 as a signature cytokine, which appears to promote inflammation by stimulating the growth of the hematopoietic cells and the mast cells and synthesize chemokines (100). Tfh cells are characterized by a high expression of the T-cell activation marker programmed cell death-1 (PD-1) and are necessary for class switching in germinal centers, high-affinity antibody generation, and B-cell maturation and differentiation (101). Tfh cells primarily localize in the lymphoid organs, but are also found in the peripheral tissues and lesions. Circulating Tfh cells comprise Tfh1, Tfh2, and Tfh17 subpopulations, which play important roles in human autoimmune diseases (102). A recent US cohort study identified that the activated circulating PD-1+ Tfh2 cells serve as a complementary biomarker of disease activity in the IgG4-related sclerosing pancreatitis and cholangitis in combination with other clinical markers, such as the plasmablasts and serum IgG4 and IgE, and as a potential target for immunotherapy based on decreases in the numbers of the PD1+ Tfh2-cell subsets after treatment, which is in line with clinical findings (103). There is a paucity of available data on the interaction among Th9 or Tfh cells and AIP or other pancreatic diseases. Further studies will be needed to determine their functions and specificity in the course of pancreatic disorders.

#### CD8+ T Cells

To date, there is no consensus about the alteration of CD8+ T cells, a subset displaying suppressor activity or cytotoxicity, in AP. One study reported no difference (25), while others reported a significant depletion or an increase in the CD8+ T cells (66, 104). Fonteh et al. suggested that these conflicting results may be due to the presence of bacterial infections caused by a second hit (105). In addition, there are variable data on the lymphocyte subsets in the circulation, the spleen, the pancreas, and main organs in relation to the timing and severity of AP. Clearly, a more efficient method or comprehensive and thoughtful experimental design will be needed to precisely detect the dynamic changes of T cells in different organs. In AIP, the infiltrating cells predominantly comprise CD4+ T cells, with few detectable CD8+ T cells. Paradoxically, one case study reported an excessive CD8+ T cell infiltration in the pancreas and extrapancreatic lesions in a 64-year-old Chinese man with AIP, indicating that AIP may have heterogeneous autoimmune origins (106). Thus, more thorough studies focusing on the role of CD8+ T cells will be required to further understand the immunopathogenic mechanisms of both AP and AIP. Tumor-specific CD8+ T_C_ cells infiltrated into tumors present a remarkable antitumor ability; however, PC is classically described as a cold or non-inflamed tumor due to a relative paucity of intratumoral CD8+ T_C_ cells. However, PC is classically described as a cold or non-inflamed tumor because of the relative paucity of the intratumoral CD8+ T_C_ cells (18). High CD8+ T-cell infiltration reportedly is an independent favorable prognostic factor associated with an improved outcome (107). Therefore, promoting CD8+ T-cell recruitment to tumors may be a major part of immunotherapy for PC.

#### γδ T Cells

The γδ T cells also are an important source of IL-17 and are involved in tissue homeostasis, infection (including bacterial and fungal), autoimmunity, cancer progression, and inflammatory disorders (108, 109). However, a few studies have reported a key pathogenic role of the γδ T cells in the inflamed pancreas. In a mouse model of the coxsackievirus B-induced pancreatitis, an overexpression of IL-17A and an expansion of the γδ T cells in the pancreas aggravated the inflammatory immunological injury to the pancreas, which was primarily induced via a pancreatic neutrophil infiltration and a peripheral Th17 response, and a γδ T-cell deficiency in a mouse model reduced the severity of pancreatitis (71). In PDAC, the γδ T cells expand more extensively than that observed in the PC, and they are localized more closely to the carcinoma cells (110). In general, the γδ T cells have been hypothesized to be antitumor entities in diverse tumor subtypes (111), even in PC (112). New evidence suggests that they are ubiquitous and that their dominant population is increased and constitutes 40−75% of the human PC-infiltrating T cells in PC (73). Blockade of PD-L1 in the γδ T cells promoted the infiltration of the immunogenic Th1 and CD8+ T cells, indicating that the γδ T cells were an important source of checkpoint ligands in PDAC. Furthermore, depletion of the intrapancreatic γδ T cells strongly protected against oncogenesis *in vivo* and led to an influx of the immunogenic Th1 and CD8+ T cells into the TME, which inhibited pancreatic oncogenesis and enhanced survival in the human PC (113). Thus, the γδ T cells appear to be key regulators of an effector T-cell activation in PC and a new target for cancer immunotherapy.

## Regulatory Mechanisms of T-Cell Alterations

### T-Cell Activation and Differentiation

There is good evidence that members of the mitogen-activated protein kinase superfamily contribute to lymphocyte activation, cytokine production, differentiation, and apoptosis (114). The CD4+ and the CD8+ T cells in SAP patients have been found to exhibit impaired NF-κB activation, which increases the risk of infection, and markedly enhanced the p38 activation, which sustains inflammation (115). A number of excellent studies have confirmed that the STAT proteins play fundamental roles in the polarization of Th1, Th2, and Th17 cells, and STAT1, STAT6, and STAT3 support these lineages, respectively. An impaired STAT1 activation combined with an enhanced STAT6 activation indicates a shift from Th1 to Th2. STAT3 is an important determinant of the differentiation of naive T cells into either Th17 or the inducible-Treg cells (116). IL-10 is thought to regulate innate and adaptive Th1 and Th2 responses by acting as an anti-inflammatory cytokine and exerting a suppressive function via Tregs (117). However, in the development of a chronic inflammatory disease, IL-10 was found to play an opposite role (27). In the pancreatic tissue, an excessive IL-10 production is pathogenic and related to the altered kinetics of Treg responses, which can promote the development of SAP and facilitate the progression of chronic disease due to the delayed innate and T-cell responses (80).

**Figure 2:**
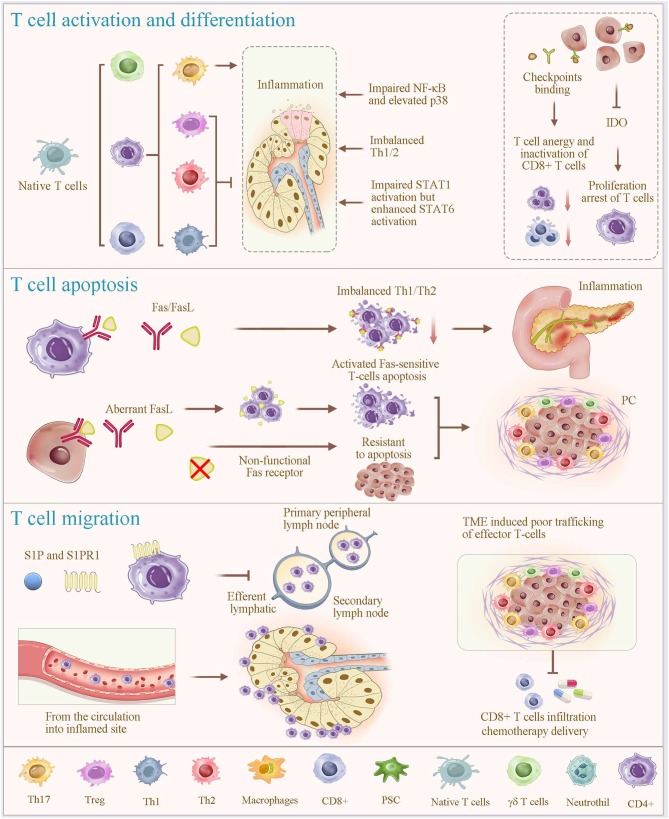
Regulatory mechanisms of T-cell alterations. T-cell programming, including activation, differentiation, apoptosis and migration (homing), plays a vital role in regulating T cell alteration. Different subsets of T cells are differentiated from naive T cells under specific stimulatory condition (most prominently cytokines). In which, members of the mitogen-activated protein kinase superfamily contribute to the process of lymphocyte activation, cytokine production, differentiation and apoptosis; and the STAT proteins play fundamental roles in the polarization of Th1, Th2, and Th17 cells, and STAT1, STAT6, and STAT3 support these lineages, respectively. Overexpression of IDO promotes proliferation arrest of T cells. Fas signaling is considered a key mechanism of immune cell apoptosis. Aberrant expression of functional Fas ligands due to nonfunctional Fas receptors of PC cells, induces activated Fas-sensitive T cell apoptosis. Immune checkpoint binding of T cells in PC results in a T-cell anergy and an inactivation of the T_C_ cells. Notably, the functions of T cells in infection control, autoimmunity, and tumor eradication involve T-cell homing. The interaction between sphingosine-1-phosphate (S1P) and its receptor S1PR1 enables lymphocyte homing, contributing to the rapid accumulation of recirculating cells. Together, significant T-cell depletion after inflammation is associated with an excessive apoptosis, migration to the local inflamed tissues, and lymphocyte homing. This physiological process is shared and used by cancer cells, which orchestrate immune exclusion as an important part of their immune suppressive strategy. IDO, indoleamine 2,3-dioxygenase; S1P, Sphingosine-1-phosphate.

T cells in PC strongly express PD-1, whereas PC cells increase the expression of the immune inhibitory ligands as well as the immune checkpoints (PD-L1 and the T_C_ cell-associated protein 4), which likely results in a T-cell anergy and an inactivation of the T_C_ cells (7, 118). Additionally, metastatic PC cells up-regulate the production of indoleamine 2,3-dioxygenase, an enzyme that catalyzes tryptophan degradation, resulting in the arrest of proliferation of T cells under a tryptophan shortage. In particular, indoleamine 2,3-dioxygenase increases Tregs in the lymph nodes, which leads to an immune evasion (119). In addition, a tumor-induced reduction in the conventional dendritic cell 1 development limits the direct T-cell activation by cancer antigens, which mediates impaired antitumor CD8+ T-cell responses and leads to the loss of control of tumor progression (Figure 2) (120).

### T-Cell Apoptosis

Fas signaling is considered a key mechanism of immune cell apoptosis. Clinical research has shown that overexpression of Fas caused excessive apoptosis of T cells and sharp drops in CD4+ T cells and the CD4+/CD8+ ratio in the peripheral blood in SAP, particularly when accompanied by sepsis. Therefore, Fas expression is negatively related to the severity and immune status of AP (121), which is consistent with findings in an experimental model of AP (122). In experimental SAP, Fas/Fas ligand overexpression is tightly related to infectious complications and disease severity via promoting T-cell apoptosis. Furthermore, immune evasion of PC cells is possibly mediated by the FasR/FasL system, including nonfunctional Fas receptors, resulting in their resistance to apoptosis and aberrant expression of functional Fas ligands inducing activated Fas-sensitive T-cell apoptosis.

### T-Cell Migration (Homing)

T cell-mediated immune responses require T cells to exit from the thymus and to travel to the secondary lymphoid organs (mainly the spleen, the lymph nodes, and the Peyer patches). Subsequently, T cells return to the circulation, traffic into tissues, and encounter infectious and other antigens after activation in the lymphoid organs. The functions of T cells in infection control, autoimmunity, and tumor eradication involve T-cell homing, which is intrinsically coupled with T-cell mobility, residency, and egress (123). T-cell trafficking from the circulation into the inflamed sites soon after an initial inflammatory insult involves three steps, including the selectin-mediated rolling, the chemokine-dependent activation, and the integrin-mediated arrest (124, 125). Concurrently, T-cell egress from the inflamed lymph node is inhibited through the interaction between sphingosine-1-phosphate (S1P) and its receptor S1PR1, which enables lymphocyte homing, contributing to the rapid accumulation of recirculating cells (126). Such inhibition of the T-cell exit results in a dramatic decrease in the number of lymphoid cells, especially T cells, in the peripheral blood. In the inflamed tissues, T cells are intrinsically programmed for antigen detection, suggesting the nature of their protective functions against inflammation. Noteworthy, it is currently recognized that imbalances in the T-cell subsets, such as imbalanced Th1/Th2 and Tregs/Th17 cell ratios, and an excessive procytokine secretion by these cells coordinately accelerate inflammation. Together, the above-mentioned findings indicate that significant T-cell depletion after inflammation is associated with an excessive apoptosis, migration to the local inflamed tissues, and lymphocyte homing. Subsequently, we have further dissected whether this physiological process is shared and used by cancer cells and other anti-inflammatory cell subtypes, which orchestrate the immune exclusion as an important part of their immunosuppressive strategy (125).

### The TME Suppresses Effector T-Cell Homing in PC

The TME features a dense stromal reaction, which is composed of structural components of the extracellular matrix and stroma, and infiltrating immune cells, including the Tregs and several types of myeloid-derived suppressor cells, cytokines, and chemokines. All of these factors impact cancer progression and clinical outcomes (127). The hypovascular and desmoplastic stroma has been suggested to serve as a physical barrier to T-cell infiltration and chemotherapy delivery to the tumor, resulting in therapy resistance (128, 129). Other receptors overexpressed in PC, such as chemokine receptors 2 and 5, have been considered to be involved in an immunosuppressive cell homing from the circulation into the TME (130). In addition, the macrophages, the cancer-associated fibroblasts, and the activated pancreatic stellate cells also inhibit T_C_-cell infiltration and exclude the T cells from the tumor (131, 132). As described above, all of these cells contribute to a more favorable TME to suppress the CD8+ T_C_-cell infiltration and subvert an immune surveillance, thereby supporting tumor proliferation, invasion, and metastasis (133).

## Treatment Options

### S1P Receptor Agonists

FTY720 (fingolimod) is a derivative of myriocin and a synthetic analog of S1P. Administration of FTY720 with or without rapamycin effectively suppressed the development of pancreatic necrosis and ameliorated the severity of SAP through an early suppression of the Th cells and by reducing the infiltration of CD4+ and CD8+ T cells in the pancreas (134). In addition to the protective effects against AP, FTY720 treatment markedly improved CP via prevention of a pancreatic inflammation and fibrosis through inhibition of the infiltration of CD4+ and CD8+ T cells, and reduction of the expression of interferon and TGF-β1 in the pancreas (135). Notably, as a nonselective agonist of S1P receptors (except S1P2), FTY720 showed an evident disadvantage of a transient dose-dependent bradycardia and mild hypertension due to the activation of the type-3 S1P receptors in phase III clinical trials, suggesting that cardiovascular monitoring at first administration is essential (136).

SEW2871 is a selective type-1 S1P receptor agonist that specifically and effectively acts on the S1P receptor, but not on the S1P2–5 receptors in humans and mice (137). A recent study demonstrated that SEW2871 ameliorates the severity of the caerulein-induced AP in mice, partly through depletion of peripheral CD4+ T cells and a reduction in CD4+ T-cell infiltration in the pancreas (77). Thus, pharmacological agents targeting S1P signaling provide therapeutic benefits by reducing the infiltration of lymphocytes into the circulation and tissue lesions, showing that this general approach is and increasingly will be important to the field (Table 1).

**Table 1:** Overview of pharmacological strategies targeting T cells in pancreatitis and pancreatic cancer.

**Category**	**Drugs**	**Targets**	**Mechanism**	**Current status**	**Application**	**Reference**
S1P receptor agonist	FTY720	S1PR1,3,4,5	T cells trafficking/homing	Clinical trials	AP, CP	(134–136)
	SEW2871	S1PR1	T cells trafficking/homing	Preclinical	AP	(77, 137)
Immunosuppressive drugs	Tacrolimus	Calcineurin	Inhibit T cells activation	Clinical	CP, AIP	(138–141)
	Rapamycin	IL-2	Inhibit effector T cells and promote Treg	Clinical	AIP	(142, 143)
	Cyclosporine A	Calcineurin	Inhibit effector T cells	Clinical	AIP	(144)
Cholinergic agonist	Nicotine	Selective cholinergic receptor	Enhanced the number and suppressive capacity of Treg	Preclinical	AP	(77)
Multifunctional cytokines	Interferon α	—	Inhibit Th17 cells and promote Treg	Clinical	AP	(145, 146)
	Thymosin α1	—	Regulating differentiation of CD3/CD4+ T cells; Balancing CD3/CD4+/CD8+ T cells in peripheral	Preclinical	AP	(147, 148)
Stem cell therapy	—	—	Decreased T-cell infiltration and enhanced recruitment of Treg	Preclinical	AP, CP	(149–154)
Hydrogen therapy	—	—	Restoring the Treg loss	Preclinical	AP, CP	(155, 156)
CAR T cells therapy	—	—	Genetically engineered T cells	Preclinical	PC	(157–164)

*AP, acute pancreatitis; CP, chronic pancreatitis; AIP, autoimmune pancreatitis*.

### Immunosuppressive Drugs

#### Tacrolimus

The macrolide immunosuppressant tacrolimus, also known as FK-506 or its trade names Prograf and Advagraf, binds to FKBP-12 (an immunophilin responsible for signal transduction) and then forms a complex with Ca^2+^, calmodulin, and calcineurin to suppress the action of nuclear factor of the activated T cells (138). Thus, tacrolimus is a topical inhibitor of calcineurin, a protein phosphatase that is essential for the T-cell activation (139). A recent study has demonstrated that tacrolimus has a preventive effect on CP in male Wistar Bonn/Kobori rats through inhibition of the acinar cell apoptosis and abnormal infiltration of CD4+ and CD8+ T cells. Additionally, FK506 effectively suppressed the development of AIP through augmentation of the infiltrated T-cell apoptosis by inhibiting Bcl-2; but not Bax (140). However, Ito et al. found that FK-506 could induce AP at therapeutic doses via elevation of an abnormal secretion of the pancreatic enzymes when the pancreas is overstimulated (141). Together, these findings indicate that tacrolimus can be selectively used for CP and AIP, but not for AP.

#### Rapamycin and Cyclosporine A

The mTOR antagonist rapamycin (sirolimus) is a macrolide immunosuppressant that targets a wide range of immune cell types. It exerts potent immunosuppressive effects through two mechanisms (142): (i) it inhibits the G0/G1 transition in effector T cells by blocking the translation of IL-2, and (ii) it promotes an increase in the immunosuppressive Tregs by influencing the PI3K-AKT-mTOR pathway upon T cell-receptor binding and contributing to the generation of Tregs by counteracting molecular brakes on Foxp3 induction (143). In MRL/Mp mice, rapamycin improves the course of AIP via selective expansion of Tregs and subsequent suppression of the effector T-cell response. The immunomodulatory agent cyclosporine A, a calcineurin inhibitor, has been used for the treatment of AIP in humans and has shown beneficial effects in reducing the disease severity by inhibiting the activation and proliferation of effector T cells (144). Noteworthy, both cyclosporine A and rapamycin are promising candidates for patients with AIP who are intolerant to steroids or relapse following a steroid withdrawal.

### Cholinergic Agonist: Nicotine

Nicotine is well known as the primary addictive agent in tobacco products. However, nicotine stimulation at a dose of 100−300 μg/kg enhanced the number and suppressive capacity of Tregs in SAP via induction of the expression of immunoregulatory molecules and TGF-β1 secretion (77). These results suggest that selective agonists may serve as an immunotherapy for SAP and AIP. Given the limitation posed by nicotine addiction, more high-quality experimental research and clinical trials will be needed to assess the suitable dose for a pancreatic treatment.

### Multifunctional Cytokines

The multifunctional cytokines IFN-α and thymosin alpha 1 have been tested for the treatment of AP. IFN-α up-regulates IL-10 production in the activated CD4+ T cells, thus exerting a profound anti-inflammatory effect through extending the negative-feedback mechanism ascribed to IL-10. In addition, its anti-inflammatory reaction is implicated in the inhibition of Th17 cell development and the stimulation of Treg function (145, 146). Thymosin alpha 1 is a polypeptide hormone produced by the thymic stromal cells that enhances the effector T-cell response by stimulating the T-cell differentiation and maturation and promoting a proinflammatory cytokine release (147). Yao et al. demonstrated that it protected against and improved the severity of SAP in rats by regulating the differentiation of the CD3/CD4+ T cells, balancing the CD3/CD4+/CD8+ T cells in the periphery, and reducing the release of serum cytokines (148).

### Stem-Cell Therapy

Mesenchymal stem cells (MSCs) have attracted substantial attention in the therapeutic setting for tissue repair and immunomodulation, and they show potential for injury repair in AP. Transplanted human MSCs markedly blocked the production of proinflammatory cytokines and suppressed inflammatory damage to the pancreatic tissue in a rodent model of SAP (149, 150), which was largely attributed to a decreased T-cell infiltration and an enhanced Treg recruitment (165). MSCs protect against CP by suppressing inflammation, partly through inhibition of the T-cell proliferation (151, 152). Noteworthy, MSCs derived from CP patients possess immunomodulatory and prosurvival capacities comparable to those of MSCs from healthy donors (153). Thus, MSCs can be effective as a novel cell-based therapeutic approach by autologous cell therapy in AP and CP. Animal trials have revealed the therapeutic potential of MSCs and their safety and efficacy, and follow-up studies to investigate their precise mechanism and human clinical trials to evaluate their therapeutic efficacy in AP and CP are warranted (154).

### Hydrogen Therapy

Molecular hydrogen (H_2_) has been widely acknowledged as a potent free radical scavenger. It rapidly diffuses across the cell membrane and selectively scavenges toxic free radicals, such as hydroxyl radicals and peroxynitrite (155). Chen et al. found that hydrogen treatment restored the loss of Tregs by promoting Treg survival and blocked the generation of reactive oxygen species in Tregs in CP, and the protective effect of hydrogen was abolished upon Treg depletion (156). Thus, hydrogen improves multiple symptoms of pancreatitis by exerting various effects on T cells.

### Chimeric Antigen Receptor (CAR) T-Cell Therapy

Tumor resection is the only potentially curative treatment for PC; however, 80% of the patients are ineligible for resection because they are diagnosed at an advanced stage, and because of the dismal prognosis, an early metastatic dissemination, and tumor resistance to the conventional treatments (chemotherapy, targeted therapy, and radiotherapy), the five-year survival rate is only 5–7% (166, 167). PC is characterized by an immunosuppressive microenvironment, which involves the CD8+ T-cell exhaustion and the infiltration of tumor-promoting immune cells. The tumor immune system has prognostic immune signatures of predicting survival and provides a robust basis for immunotherapy in PC (168). However, the response to single-agent immune checkpoint inhibitor (CPI) therapy is unsatisfactory in PDAC (169). Therefore, there is an increasing interest in combination therapies and novel immunotherapy strategies for PDAC, such as the CAR T-cell therapy, which may reverse an immune escape and activate immune cells targeting cancer antigens (170). CAR T-cell therapy is a novel adoptive T-cell therapeutic modality in which engineered T cells that express the synthetic tumor-associated antigen receptors are expanded *ex vivo* and then adoptively reinfused into patients. By specifically targeting the tumor-associated antigens, the CAR T cells, which have immunologic memory, eradicate tumor burden via T-cell activation and cancer-cell lysis (157, 158). CAR T-cell therapy is considered a potentially valuable strategy for evaluating immune resistance mechanisms in PC (159). The evolution of CAR designs includes three generations to date. Ongoing preclinical studies focus on various cancer cell-surface antigens, such as mesothelin, the carcinoembryonic antigen, MUC1, the prostate stem cell antigen, CD24, HER2, and the natural killer receptors (158, 171). MUC1-CAR T cells, which are second generation, have recently been shown to have potent cytotoxicity against cancer cells and to induce tumor regression *in vivo* (160). In a study aimed at improving inadequate intratumoral T-cell trafficking, Jin et al. demonstrated that IL-8 receptor modification significantly promoted the trafficking and intratumoral persistence of the CAR T cells, resulting in a maximal antitumor response by inhibiting tumor growth and forming a long-lasting immunologic memory to reverse tumor immunosuppression (161). In order to overcome antigen heterogeneity and improve antitumor response efficacy by modulating CAR expression levels and T-cell affinity, robust CAR designs, such as dual tumor-associated antigen-targeted, tandem, and switchable CARs are currently under investigation (172). The broad CAR-antigen landscape will potentially provide a revolutionary treatment for PC.

In addition to CPIs, other single-agent immunotherapies (e.g., vaccines, oncolytic viruses, and TGF-β inhibitors) even when combined with gemcitabine chemotherapy, failed to induce a therapeutic response in PDAC, implying that PDAC was resistant to monotherapies (37). Therefore, current studies are mainly focused on combination therapies (173). For example, combination therapy with CPIs reportedly improved the potency of CAR T cells (174, 175). In addition, novel combinations of CPIs with the mesothelin-targeted or the MUC1-expressing CAR T cells are actively investigated (162).

As most potential CAR T-cell targets in solid tumors are also expressed in some healthy tissues, off-tumor toxicity, including neurotoxicity, cytokine release syndrome, and the CAR-related encephalopathy syndrome, remains an obstacle for the clinical development of CAR T-cell therapy (176, 177). However, studies continue to assess the safety of CAR T cells. For example, Shivani et al. demonstrated that the use of combinatorial antigen sensing circuits using the synthetic Notch receptors can result in tumor regression without an off-tumor toxicity when tumor and healthy tissues are not highly colocalized (163). In a phase I study of the anti-mesothelin CAR T cells in PDAC patients, the therapeutic cells showed an acceptable safety, and encouraging clinical activity (164). The safety concerns with CAR T cells were acceptable, and the efficacy was encouraging. Based on the dynamic and reciprocal regulation of the PC TME and CAR T cells, integrated immunotherapies aimed at overcoming the therapeutic resistance of PC will provide new opportunities. We remain hopeful that powerful approaches will be designed for improving the safety of the CAR T-cell therapy by avoiding its serious adverse events. Further, the identification of predictive biomarkers to guide patient selection for CAR T-cell therapy deserves continuous research efforts (37).

## Concluding Remarks

In this review, we concentrated on the mechanisms underlying the alteration of T cells in pancreatitis and PC, which are attributed to various aberrations in T-cell programming, including activation, differentiation, apoptosis, and migration (homing). Prospective monitoring of the T-cell alterations may aid in predicting the clinical outcome of pancreatic inflammation. Collectively, immunomodulatory approaches targeting an excessive infiltration of T cells, balancing the Th1/Th2 cells and the Tregs/Th17cells, and restoring the Treg loss are useful in halting the disease progression by preventing a pancreatic inflammation. Notably, the actual function and change in the CD8+ T cells during pancreatic inflammation remain unclear and require further study to gain deeper insights in these aspects. T-cell homing deficits mediated by the TME render traditional immunotherapies inefficient. Novel strategies targeting T cells, especially the local administration of the CAR-T cells and combination with other immunotherapies, provide a novel opportunity for patients with PC, given the multitude of targets. Taken together, immunotherapies targeting the T cells may have important implications for inflammatory diseases and cancer in the pancreas.

## Author Contributions

QZ and XT conceived this review and drafted the manuscript. SX, FG, and CP consistently contributed references as well as drew the figures. HX edited and finalized the manuscript for submission. DS reviewed and approved the submitted manuscript.

### Conflict of Interest

The authors declare that the research was conducted in the absence of any commercial or financial relationships that could be construed as a potential conflict of interest.

## Abbreviations

AP: acute pancreatitis
AIP: autoimmune pancreatitis
CAR: chimeric antigen receptor
CP: chronic pancreatitis
CPI: checkpoint inhibitor
IFN: interferon
IPMN: intraductal papillary mucinous neoplasm
MSC: mesenchymal stem cell
PanIN: pancreatic intraepithelial neoplasia
PC: pancreatic cancer
PD-1: programmed cell death-1
PDAC: pancreatic ductal adenocarcinoma
S1P: sphingosine-1-phosphate
SAP: severe acute pancreatitis
SIRS: systemic inflammatory response syndrome
STAT: signal transducer and activator of transcription
T_C_ cell: cytotoxic T cell
Th: T helper
TME: tumor microenvironment
Treg: regulatory T
Tfh: T follicular helper.
